# Lectures on General Physiology

**Published:** 1855-11

**Authors:** 


### ECLECTIC DEPARTMENT,

### AND SPIRIT OF THE MEDICAL PERIODICAL PRESS.

---

### *Lectures on General Physiology.* By CLAUDE BERNARD.

SUMMARY: Secretion—Cells—Secretion independent of nervous action—Experiment demonstrating the independence of cell action—Products of secretion—Two orders of secretions—Non-absorption of secretions—Hepatic sugar—Distinctions of secretions and excretions.

In studying the chemical phenomena which takes place in the economy, the property of secretion, or that aptitude which certain tissues possess for producing certain liquids, is one of the most important of the functions offered for our consideration.

Herein, especially, we shall perceive how the chemical phenomena of living beings present characteristics which are not observed elsewhere; how they differ from analogous phenomena out of the organism; and form, as it were, a connecting chain between living beings and the inorganic world.

The property of secretion belongs, in general, to all things endowed with life, animals and vegetables; the tissue in which it is accomplished may always be reduced to the simple cell.

Whatever the animal or vegetable examined; whatever the character of the secreted product, we always find this cell; which proves that the form of a tissue is not always sufficient to indicate its function.

As I said that the fibre was the essential element of contractile tissue, so I can say that in the cell resides the property of secretion. The cell consists of two parts; the envelope or cell-wall and the nucleus. Physiologists have discussed whether the property of secretion resided in the envelope or in the nucleus. To us this problem is of slight interest. We may be satisfied to know that the cell creates certain products. The cell has the property of maintaining its suitable organic condition, by attracting certain substances from the medium by which it is surrounded. It secerns certain elements from this medium, and so acts on them that they leave it under a different form. This is a creation, not of elements, but of the form in which they are aggregated. Albumen is transformed into sugar; the transformation is the work of the glandular cell. I have said that this property is inherent in the cell as contractility is inherent in the muscular fibre. The nervous system reäcts on both these tissues. It does not endow them with their properties, but it sets them in action. The *woorara* destroys the properties of the nerves, but it spares the muscles, as we have shown (Vol. iii., p. 498, *experiment.*) Glands also escape its influence. If we poison a leech by woorara, and replace the stimulus of the nerves, we shall find the secretion of mucus going on as if the nervous system was in full activity.

[The professor here exhibited a leech that had been poisoned on the preceding night. It was motionless and incapable of motion. The *galvanic forceps* was then applied to its body, and immediately mucus was observed, abundantly secreted, and flowing toward the two extremities of the forceps. This was the first occasion on which M. Bernard had exhibited this experiment in public.]

If we admitted that the nerves endowed glands with the property of secretion, we should be forced to admit as many different varieties of nerves as there are varieties in the products of secretion. The nervous system, then, is an exciting agent. The glands are independent, as to their property of secretion; as to the *time at which they secrete*, they depend on the nervous system.

The products of secretion, in the two kingdoms, are various. Sometimes in animals, they are a species of new agents, which we call *fermenting principles.* The glands of the stomach, for example, secrete a liquid, the gastric juice, composed of acidulated water and *pepsine;* the pepsine is a fermenting agent. Farther on, the pancreatic juice is secreted, which is a chemical agent. Poisons are secreted, both in the animal and vegetable kingdoms. Some serpents secrete active poisons, analogous to ferments in their action. In the animal, secretion is influenced by nervous power; in the vegetable, it is affected by general atmospheric and other agencies. Analogous products, nevertheless, are found in the animal cell and in the vegetable cell. Starch and sugar are vegetable products, but the liver also produces sugar, as the pancreas secretes the pancreatic juice, and the stomach secretes pepsine. Albuminoid matters, also, are secreted by vegetables; milk, an animal product, is composed of caseine and fat.

As secretion takes place, we see the product deposited in the cell; then the cell breaks up, and the product is discharged. I cannot pretend to enumerate, at present, all the products of secretion. What it is important to know is, that these products are derived from the cell itself,—that *the glands produce and are not mere agents of elimination.* According to old theories all products of secretion were contained originally in the blood, and the glands only separated and excreted them. This notion is entirely erroneous. The *elements* of secretions are in the blood, not the secretions themselves. The hepatic cell finds the elements of sugar in the blood, but it does not find sugar there.

I make two orders of secretions:

I. *Intestinal Secretions.* Pepsine is produced by the cells of the stomach; it is not found in the blood. The blood which enters the pancreas does not contain poncreatic fluid; the elements of the secretion are there; the blood contains oxygen, hydrogen, carbon, nitrogen, etc., but the perfect product is not contained in the blood. The venifacient glands of serpents furnish another, and a striking, example of this truth. Is the poisonous product contained in the blood of the serpent? Surely not; and if his own venom is introduced into his circulation, the animal dies.

Not only are these products of secretion, not to be found in the blood, but they cannot enter the circulation except by artificial means. If the serpent swallows his own venom, he does not die. Savages eat the flesh of animals killed by the woorara. The South American Indians use woorara as a medicine, as M. de Humboldt informs us. The poison cannot be absorbed by the intestine. Take a dog, and introduce woorara into his stomach,—he suffers no harm thereby. Introduce the same poison in a vein, and he dies instantly.

The general characteristic of all products secreted in the intestinal tube, is that they are not reäbsorbed in the digestive canal. If it was otherwise, these products would have no time to act on substances coming from without, which they are designed to modify. The digestive tube is a closed cavity as far as these products are concerned. We must abandon the old ideas, therefore, knowing that these products of secretion are not formed originally in the blood, and that they cannot return into the circulation after they are formed.

II. There are other products of secretion, which are poured into the blood, and which do not leave the circulation. I only speak now of what takes place in animals. It is to be regretted that this question has not been studied as regards the vegetable kingdom, and that general physiology is not more advanced on this point. I desire, however, to treat only of what is really ascertained, and shall confine myself to the narrow limits of positive facts. The products I now speak of are called *immediate products.* The liver, for example, secretes sugar in all vertebrate animals, and in many vertebrata; an hepatic cell, bathed in blood destitute of sugar, selects from the blood the elements of its secretion, and groups them under the form of sugar. We can demonstrate that the blood that enters the liver contains no sugar, whereas sugar is constantly present in blood issuing from the liver; the blood that has passed through the liver, is, moreover, deficient in albumen and fibrine; whence we may conclude, that it is at the expense of these two substances that sugar is formed. Here, again, there is a nerve that influences the cell, and we may increase or diminish the secretion of sugar by stimulating this nerve more or less. In vertebrates, the sugar is not emptied into an excretory duct, it enters the blood immediately: the veins, in fact, are the excretory ducts of the liver. Moreover, the sugar is not separated from the blood; it remains in it to subserve the purposes of respiration. This, then, is an example of a secretion emptied into the blood. I should mention, however, that in invertebrates, the sugar leaves the blood and is reäbsorbed. It is carried into the intestine by the choledoch duct, which is alternately a canal for sugar and bile. This arrangement is made necessary by the differences in the circulation of these animals.*

There are many products of secretion in the same category with sugar, with which we are as yet unacquainted. In former times, nothing was considered a secretion unless it issued from an excretory duct. We now know that secretions occur where there is no excretory canal. It is in this way, in all probability, that the thyroid body, the supra-renal capsules, the spleen, and some other organs, modify the blood. We do not know their products as yet, but sooner or later, they will be discovered. Perhaps albumen and fibrine are secreted and poured into the blood in like manner as sugar.

We make, then, two orders of secreted products:

---

* In the number of this journal for August, 1855, (vol. I, p. 343,) Dr. H. L. Thomas published a translation of a very complete paper on the secretion of sugar by the liver, by Dr. Vernois, in which the subject here referred to is treated in detail.

1. Those which are emptied without the circulation and are not reäbsorbed into it.

2. Those which are emptied into the blood, and do not leave it.

If products of the second class leave the blood, it is in consequence of some abnormal state, as, for example, diabetes or glucosuria.

Physiologists for a long time, confounded excretion with secretion. And it was chiefly with arguments based on the urinary excretion, that the doctrine of the products of glands existing primarily in the blood was maintained. The following experiment was adduced in support of this opinion. Urine was analyzed and found to contain urea and uric acid. It was then asked, whether the kidney secreted these products as the stomach secretes pepsine.

Prévôt and Dumas devised an experiment to determine this problem. They removed the kidneys of dogs, and on analyzing the blood subsequently, they found in it urea and uric acid. It was naturally concluded, that these products were formed in the blood and by the kidneys. By an erroneous idea generalization has been based on this fact. It has been asserted that glands are not *formative*, but exclusively eliminative in their action. Now, what is true of the kidneys, is not true of the glands. Pressure exerts no influence on the secretion of glands,* but a very great influence on the urinary excretion.

A product of *secretion* is a substance elaborated for a certain purpose; pepsine, pancreatic fluid, sugar, and venoms; have certain peculiar uses. A product of *excretion* is no longer of any use in the economy; its presence is deleterious. Carbonic acid is not elaborated anywhere in the organism. It is produced by the decomposition of hydro-carbonaceous materials. Uric acid results from the decomposition of azotized matters. The lungs eliminate the carbonic acid; the kidneys expel the urea and uric acid.

The secretions are always effected by special organs, and cannot take place elsewhere; the excretions, in the failure of one organ, can be accomplished by another. This vicarious action is remarkable in dogs, and remained unexplained for a long time.

Urea accumulates in the blood, when the kidneys are removed, as I have already stated; but after a short time has elapsed, only a small proportion of it is found in the blood, for it is eliminated in a great measure by the intestinal canal. This is an example of a displaced excretion. The digestive tube takes the place of the renal system. The system throws off, by a different channel, the injurious products, that would otherwise accumulate in the blood. If you cut the nerves of the stomach, the secretion of gastric juice will cease, and nothing will replace it. There may be metastases of excretions, but never of secretions.

Excretion cannot, therefore, be confounded with secretions.—*Virginia Med. and Surg. Journal.*

---

*On the Glucogenetic Functions of the Liver.*—It has been generally acknowledged, for some years past, that Bernard's discovery of the formation

---

* See the experiment in the Seventh Lecture. Vol. V., p. 125.

